# CRP, but not fibrinogen, is associated with gait speed as early as middle age, in females but not males

**DOI:** 10.1038/s41598-023-42183-1

**Published:** 2023-09-20

**Authors:** Noha Shekh Ahmed-Yousef, Omer Dilian, Khalil Iktilat, Maayan Agmon

**Affiliations:** The Cheryl Spencer Department of Nursing, Faculty of Social Healthcare and Health Sciences, University of Haifa, Haifa, Israel

**Keywords:** Predictive markers, Geriatrics

## Abstract

Low grade systemic inflammation and age-related gait speed decline are known to be related in older adults, but their relations in the early stages of the aging process are yet to be fully described. The aim of this study was to examine the relationship between gait speed and two inflammation markers—c-reactive protein (CRP) and fibrinogen—in a cohort of middle-aged adults in Israel. 326 healthy, middle-aged, Muslim-Arabs from three villages in northern Israel participated in this cross-sectional study. Serum CRP and fibrinogen were measured via blood tests, and gait speed was assessed with the 6-min walk test (6MWT). After adjusting for sex, age, height, BMI, systolic blood pressure, fasting blood glucose and triglycerides, executive function, smoking status and aerobic physical activity, gait speed was negatively and significantly associated with CRP (b = − 0.01, p = 0.029). When stratifying by gender, this link remained significant only among females (b = − 0.012, p = 0.041), such as that an increase of one SD unit of CRP was associated with a 0.047 m/s decrease in gait speed. No significant link was found between fibrinogen levels and gait speed. Blood CRP levels are associated with a slower walking speed already in middle age, independent of age, executive function and cardio-metabolic factors, among female Arab-Muslims in Israel. Future studies should examine this relationship longitudinally and investigate a broader array of inflammation markers. Systemic inflammation may serve as an early marker for people at risk of decreased walking or accelerated aging; Early identification and intervention among at-risk individuals may help prevent or slow gait speed decline, and promote healthier aging.

## Introduction

The world's population is rapidly aging, and the identification of risk factors for accelerated aging and promotion of healthy aging have been proposed as major public health goals^1^. Mobility, defined as the ability to move independently^2^, is considered a hallmark of functional aging^3^, and decreased mobility has been linked to lower quality of life and adverse health outcomes such as falls^4^, dementia, disability and mortality, among older adults^5^. Mobility is often proxied by gait speed, seen as a simplified measure predicting adverse health effects and functional decline.^6,7^

Gait speed gradually declines with aging, a process that is thought to be related, among other factors, to low-grade systemic inflammation—seen by increased inflammation markers in serum characteristic of old age^8–10^. It is hypothesized that increased inflammation could mediate age-related gait decline^9,11^, with higher levels of inflammatory markers such as C-reactive protein (CRP), fibrinogen, IL-6 and TNF-α being associated with decreased gait speed among older adults in multiple studies^8,10–13^. However, this association has been very rarely examined in middle-aged adults.

In recent years, it has become clear that gait deterioration, among other age-related processes, begins as early as middle age^14,15^. Identification at this preliminary stage could allow for treatment adjustment and the development of appropriate interventions^9^. The few studies conducted on middle-aged populations present conflicting results as to whether increased inflammation markers are associated with decreased gait speed as early as mid-life^9,16–18^. Perhaps this inconsistency could be explained by the methodologies used to assess gait speed, with short (8–15 m) walking trails or one-minute speed tests possibly being unsuitable to measure gait speed decline in mid-life. Longer walking tests are more ecologically relevant, and recent understandings suggest that they may reflect gait-speed and physical capacity more accurately^19,20^.

This cross-sectional study examined the association between inflammatory markers (CRP and fibrinogen) and gait speed in a six-minute walk test, in a cohort of middle-aged Arabs in Israel, an understudied population. While this relationship has been studied extensively at older ages, very few past studies have examined it in middle-aged adults. Determining the relationship between inflammatory markers and reduced gait speed in early stages could improve our understanding of the pathways behind physiological and pathological aging processes, and could facilitate the implementation of interventions to slow or prevent age-related gait decline.

## Methods

### Study population

#### Sample

The study used data collected in a survey examining health-related behaviors in middle-aged Arab-Muslims in Israel. Participants were recruited from three Arab villages in northern Israel, via recruitment posters in public places, by word of mouth within each village, and by snowball sampling.

##### Inclusion criteria

- Muslims of both genders
- Aged 40–60 years
- Do not use assistive devices in walking, and without orthopedic limitations or history of lower limb injury or surgery, according to self-report.
- Without medical history that prevents participation in the study, such as hearing impairment, dementia or mental or intellectual disability, according to self-report.

##### Exclusion criteria

- Participants with CRP above 20 MG/dl were excluded, to eliminate cases of acute inflammation or illness.
- Participants with partial blood test results were excluded from the study.

### Procedure

Each participant participated in two sessions, held in public institutions such as schools and local associations. During the first session, lasting about an hour and occurring in the timeframe of 10:00–19:00, participants were asked to sign an informed consent form for their participation after the purpose of the study and anonymity procedures were explained to them. Subsequently, questionnaires related to lifestyle were distributed, biometrics (blood pressure, weight, height, waist and hip circumferences) were collected, and participants were asked to undergo a series of tests that included various skills such as walking, executive and cognitive function. The second session took place a week later at a pre-arranged institution, between 6:00–8:30 AM, during which participants underwent blood tests. Participants were requested to fast for 12 h before the second research session. After data collection, the data was coded and processed using the SPSS software package.

The research was conducted in accordance with the relevant ethical guidelines and regulations; all participants provided informed consent and the study was approved by the Ethics Committee of the University of Haifa # 237/21.

### Tools

#### Gait speed in 6 min walk

Gait speed was measured using the 6-min walk test (6MWT)—a common test to assess personal functioning abilities, suitable for a wide range of populations and requiring minimal equipment^21^, that is also considered a predictor of actual physical activity^22^. Participants were asked to walk "as fast as they can without running", in a speed that would allow them to continue walking for 6-min, back and forth between two ends of a 10-m walking trail. The test was conducted indoors, on a flat floor, in halls of community centers in each of the villages. The walking trail was marked on the floor using colored tape, with additional markers every 1-m to allow for exact measurements. Total distance walked was measured for each minute separately and for the six minutes cumulatively, and divided by time walked to receive average walking speed. In the analyses, measurements from the first minute and from the total six minutes were used – the first as a representation of gait speed as measured in short walking tests, and the second as a measure of endurance, that represents walking speed under aerobic stress^23,24^.

### Inflammation markers

Fibrinogen and CRP concentrations were measured from blood samples collected after an 8–12-h overnight fast, from an antecubital vein into a 3.8% sodium citrate-containing tube, which was stored at −80 °C until assayed. The study measurements were performed under quality control supervision of established reference laboratories, and 5% blind duplicate samples were used to estimate the analytic variation within runs and over time^25^. CRP was measured using an Abbott Architect CI-4100 (Abbott, Germany), reagent no. 8-6K26-30, with a detection range of > 0.6 mg/dl. Fibrinogen was derived using prothrombin time (PT) test, with HemosIL RecombiPlasTin 2G kit (Werfen, Barcelona, Spain).

### Covariates

Age, sex, height and weight were collected, allowing for body mass index (BMI) calculation. To account for cardiometabolic covariates, glucose and triglycerides levels from 8 to 12 h fasting blood tests were measured. Additionally, blood pressure was measured using the standard electronic blood pressure device Omron M6 Comfort (model number HEM-7360-E, Omron Healthcare, Kyoto, Japan), after subjects were requested to rest for five minutes, and their legs were touching the floor and not crossed. Blood pressure was measured before the walking test, three consecutive measurements, and average systolic pressure was calculated based on these measurements. Executive function was measured using the trail making test B, (TMT-B), used to assess cognitive flexibility and executive function^26,27^. Current smoking status was collected using a direct question—"are you smoking?", with waterpipe tobacco smokers included in the smoking group for analyses. Aerobic physical activity status was also collected using a question—"are you participating in your spare time in any physical activity lasting more than 20 min straight, causing you increased pulse, rapid breathing and sweat, such as: running, quick walking, aerobic fitness, dancing, ball games, cycling, etc.?". In both cases, status was analyzed as a binary variable, with participants being described as smokers/non-smokers, and participating or not-participating in aerobic physical activity.

### Statistical analysis

Linear regression models were used to assess the association between levels of inflammatory markers and gait speed. CRP and Fibrinogen were used as independent variables, and gait speed in 1 min and 6 min were the outcome variables.

Three linear models were used; in the first (model 1), regressions were adjusted for age, sex and height. In the second (model 2), results were further adjusted to account for BMI, triglycerides levels, fasting blood glucose, and average systolic blood pressure. In the third (model 3), results were further adjusted for additional non-physiological covariates: smoking status, aerobic physical activity status, and cognitive function. Covariates were chosen based on their known relations with gait speed, physical function and inflammatory marker levels.^28–31^ P-value < 0.05 was considered as statistically significant.

## Results

**Figure 1:**
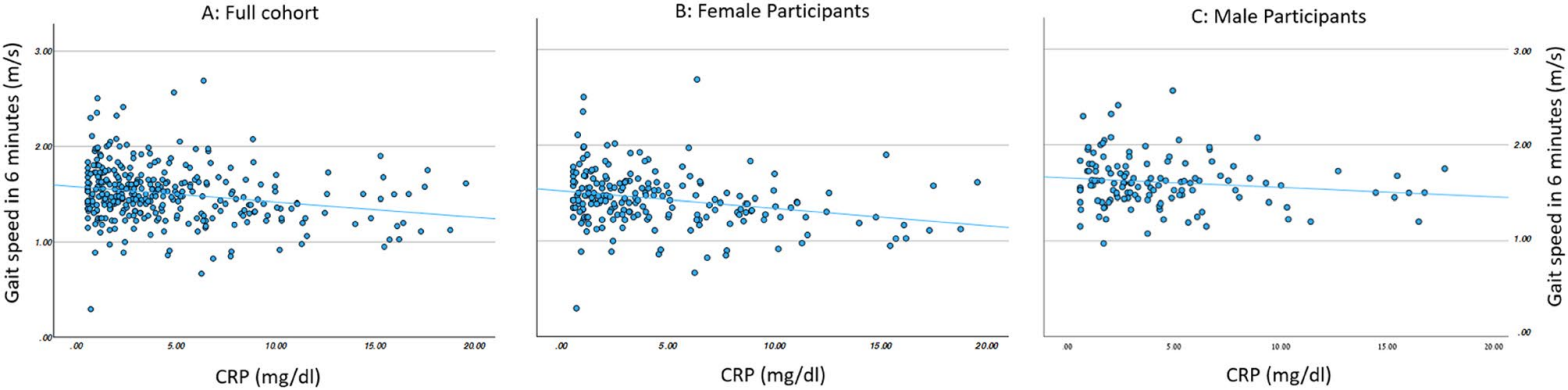
the link between c-reactive protein and gait speed in a six-minute walk. (**A**) Describes the full cohort (correlation coefficient: − 0.214, p < 0.001), (**B**) (correlation coefficient: − 0.253, p < 0.001) and (**C**) (correlation coefficient: − 0.141, p = 0.113) describe data of female and male participants respectively. All coefficients are for Pearson correlation. *CRP* c-reactive protein.

Table 1 shows the characteristics of 326 participants (61.04% female) aged 40–60 years (mean age 49.3, SD 5.35) who took part in both sessions of the study. Results are reported for the full cohort, and additionally for female and male participants separately. Independent t-tests and chi-squared tests, as appropriate, were used to compare the means of both groups, with significant differences between males and females found in smoking status, height, average systolic blood pressure, triglycerides, and both one-minute and six-minute walking speed. Figure 1A–C includes a scatterplot of the relationship between CRP and gait speed in 6-min walk in the full cohort and in the female and male groups respectively.

**Table 1:** Sample characteristics.

	All participants				Male				Female				χ^2^ p-value
N (%)	326 (100%)				127 (38.96%)				199 (61.04%)				
Regularly smoke	69 (21.2%)				55 (42.6%)				14 (7%)				** < 0.001**
Regularly participate in aerobic activity	165 (50.6%)				70 (55.1%)				95 (47.7%)				0.193
Disease prevalence and medications													
Diabetes mellitus	31 (9.5%)				16 (12.5%)				15 (7.5%)				0.128
Coronary heart disease	11 (3.3%)				5 (3.9%)				6 (3%)				0.653
Depression	7 (2.1%)				2 (1.5%)				5 (2.5%)				0.568
Medication-treated hyperlipidemia	44 (13.5%)				22 (17.3%)				22 (11%)				0.106
Medication-treated diabetes	21 (6.4%)				10 (7.9%)				11 (5.5%)				0.400
Medication-treated hypertension	45 (13.8%)				18 (14.2%)				27 (13.5%)				0.877

	Min	Max	Average	SD	Min	Max	Average	SD	Min	Max	Average	SD	Independent t test p value
Age (years)	40	60	49.3	5.35	41	60	49.93	5.142	40	60	48.89	5.450	0.085
BMI (kg/m^2^)	19.54	56.5	30.22	5.03	21.5	46.61	30.37	4.47	19.54	56.51	30.12	5.36	0.656
Height (cm)	149	194	167.83	9.14	162	194	176.19	6.24	149	188	162.57	6.32	** < 0.001**
Average systolic pressure (mmHg)	78	208.67	123.07	17.54	101.33	208.67	131.56	16.78	78	172.5	117.66	15.82	** < 0.001**
Trail Making Test (seconds)	5.46	212	87.48	33.55	5.46	212	89.67	35.03	11	192	86.06	32.56	0.342
Glucose (mg/dl)	61.08	312.37	98.99	28.25	62.77	299.98	101.81	27.95	61.08	312.37	97.18	28.36	0.147
Triglycerides (mg/dL)	36.09	756.35	134.88	83.36	45.5	756.35	166.47	108.86	36.09	356.44	114.70	53.04	** < 0.001**
CRP (mg/dL)	0.6	19.6	4.56	4.02	0.6	17.7	4.4	3.8	0.6	19.6	4.67	4.16	0.557
Fibrinogen (mg/dL)	288.5	792.3	442.68	83	288.5	792.3	432.93	77.06	309.5	777.7	448.893	86.19	0.09
Walking speed, 1st minute of 6MWT (m/s)	0.67	2.5	1.5	0.3	1	2.4	1.61	0.265	0.67	2.5	1.44	0.3	** < 0.001**
Walking speed in 6MWT (m/s)	0.29	2.69	1.5	0.3	0.97	2.57	1.61	0.265	0.29	2.69	1.44	0.3	** < 0.001**

*6MWT* 6 min walk test, *BMI* Body Mass Index, *SD* standard deviation.

Significant values are in bold.

Table 2 presents the results of the three linear regression models examining the association between inflammation markers and gait speed described earlier. The models were used to explain both gait speed in 6 min and in the first minute of the 6MWT.

**Table 2:** Regression models—whole sample.

Dependent variable	Model	Fibrinogen b (95% CI)	p	CRP b (95% CI)	p
Gait speed in 6MWT, m/s	1	0.0003 (− 0.0007, 0)	0.078	− 0.014 (− 0.022, − 0.007)	** < 0.001**
	2	0.0002 (− 0.0006, 0)	0.250	− 0.009 (− 0.018, − 0.0004)	**0.040**
	3	0.0003 (− 0.0007, 0)	0.156	− 0.01 (− 0.018, − 0.001)	**0.029**
Gait speed in 1st minute of 6MWT, meters/second	1	− 0.0003 (− 0.0007, 0)	0.083	− 0.016 (− 0.023, − 0.008)	** < 0.001**
	2	− 0.0002 (− 0.0006, 0)	0.287	− 0.009 (− 0.018, − 0.0008)	**0.032**
	3	− 0.00025 (− 0.0006, 0)	0.207	− 0.009 (− 0.018, − 0.0003)	**0.042**

Model 1 adjusted for age, sex, and height.

Model 2 further adjusted for: BMI, glucose, triglycerides and average systolic pressure.

Model 3 further adjusted for: executive function (trail making test score), smoking status, aerobic physical activity status.

Significant findings are in bold.

*CI* confidence interval.

In all models CRP was found to be significantly and negatively correlated with gait speed both in six- and one- minute walk. The link between fibrinogen and gait speed was, however, found insignificant in all models. Specifically, in the fully adjusted model, CRP was associated with lower gait speed such as that an increase of one standard deviation (SD) unit of CRP was associated with a 0.04 m/s decrease in gait speed in the six-minute test (p = 0.029), and with a 0.036 m/s decrease in gait speed in the one-minute result (p = 0.042). In all models, the link between fibrinogen and gait speed was found to be insignificant (in the fully adjusted model, b = − 0.0003, p = 0.156).

As gender was found to be a significant covariate in our first regression analysis, we conducted a further post-hoc regression analysis, divided between female and male participants, in an attempt to assess the impact of gender on the formerly described relationship. These results can be found in Table 3, which shows that while discerning by gender, the link between CRP and gait speed remains significant only among females. In the fully adjusted model, an increase of one SD unit of CRP was associated with a 0.047 m/s decrease in gait speed in the six-minute test (p = 0.041); when regressing on the first minute measurement, results bordered significance, with an increase of one SD unit of CRP associated with a 0.043 m/s decreased gait speed (p = 0.058).

**Table 3:** Regression models—separated by gender.

Dependent variable	Model	Fibrinogen b (95% CI)	p	CRP b (95% CI)	p
Males					
Gait speed in 6MWT, m/s	1	− 0.0005 (− 0.001, 0)	0.089	− 0.01 (− 0.022, 0.003)	0.125
	2	− 0.0005 (− 0.4, 0)	0.166	− 0.006 (− 0.02, 0.007)	0.342
	3	− 0.0005 (− 0.411, 0)	0.182	− 0.006 (− 0.02, 0.009)	0.434
Gait speed in 1st minute of 6MWT, m/s	1	− 0.0006 (− 0.001, 0)	**0.047**	− 0.012 (− 0.024, 0.003)	0.56
	2	− 0.0005 (− 0.001, 0.0001)	0.121	− 0.007 (− 0.02, 0.005)	0.253
	3	− 0.0005 (− 0.001, 0.0002)	0.171	− 0.005 (− 0.019, 0.008)	0.458
Females					
Gait speed in 6MWT, meters/second	1	− 0.0002 (− 0.007, 0.0002)	0.329	− 0.017 (− 0.027, − 0.007)	** < 0.001**
	2	− 0.0001 (− 0.0006, 0.0004)	0.606	− 0.011 (− 0.023, 0.0007)	0.066
	3	− 0.0002 (− 0.0007, 0.0002)	0.344	− 0.012 (− 0.023, − 0.0005)	**0.041**
Gait speed in 1st minute of 6MWT, m/s	1	− 0.002 (− 0.0007, 0.0003)	0.419	− 0.018 (− 0.028, − 0.008)	** < 0.001**
	2	− 0.0001 (− 0.0006, 0.0004)	0.696	− 0.012 (− 0.024, 0)	**0.05**
	3	− 0.0002 (− 0.0007, 0.0003)	0.439	− 0.011 (− 0.023, 0.0004)	0.058

Model 1 adjusted for age and height.

Model 2 further adjusted for: BMI, glucose, triglycerides and average systolic pressure.

Model 3 further adjusted for: executive function (trail making test score), smoking status, aerobic physical activity status.

Significant findings are in bold.

*CI* confidence interval.

## Discussion

In this cross-sectional study higher serum levels of CRP, but not fibrinogen, were associated with slower gait in a cohort of middle-aged Arab-Muslims in Israel. When stratifying by gender, this relationship remained significant only among women. These results support and broaden existing literature regarding the links between higher inflammation markers and gait speed decline^16^; the novelty of this study is the investigation of this connection in a middle-aged population. Few former studies have been conducted in this age group, and our results suggest that this link is already identifiable in early stages.

Our study identified a unique gendered pattern of the link between inflammation markers and gait speed; higher CRP was associated with decreased gait speed among women, but not men even after controlling for significant covariates such as age, BMI, cardiometabolic factors, smoking, executive function and physical activity. For several decades, it has been well documented that women and men undergo different aging patterns; women live longer, but suffer from higher morbidity and disability than men, a phenomenon which has been referred to as ‘the morbidity-mortality paradox’^32–34^. Women are also more likely to suffer from inflammaging and auto-immune diseases^35,36^.

Similar to our results, in a former study conducted among middle-aged women, fibrinogen was found to be a less robust marker for physical functioning and gait than CRP, both at baseline and after 5 years^18^. These findings could perhaps be attributed to the underlying mechanisms connecting the proteins to gait decline. Higher CRP levels are known to be caused by higher adiposity, through the secretion of IL-6^37^, while fibrinogen, in addition to being an inflammatory marker, is a key factor in hemostasis and thrombosis, and is highly associated with increased blood pressure^38,39^. While in our cohort women and men did not differ in their BMI, they could still differ in their adiposity patterns: higher visceral adiposity, more common in women than men, is known to be highly related to decreased gait speed^40^. In cohorts of older adults, who show elevated serum inflammatory markers, including CRP and fibrinogen, compared to middle aged adults, both markers were associated with decreased gait both in males and females^10,12,41–43^. This dynamic could suggest that the inflammatory stress induced by CRP and fibrinogen is significant only above a certain basal level, and that this threshold could vary between males and females, causing an earlier reaction among females. However, further research is required to affirm or dismiss such a hypothesis. In a world advancing towards gender medicine and personalized medicine^44^, it is clear that inflammaging is heavily affected by gender, and our study suggests that different inflammatory markers may be appropriate for the identification female patients in high-risk for gait decline and pathological aging in mid-life.

While we accounted for BMI, involved in the potential pathways through which CRP could affect gait, additional causal pathways exist; CRP could be related to gait decline either by direct tissue damage caused by inflammation, or by the promotion of inflammatory diseases and sarcopenia^45–47^. The patterns underlying the relationship between low grade systemic inflammation and gait speed decline are yet unclear, and our study provides an addition to existing initial understandings.

We hypothesize that the existing conflicting results regarding the links between serum inflammation markers and gait speed in middle age could be partly attributed to the walking test characteristics. Past studies examining the relationship between inflammation markers and gait speed tended to use short walking tests, such as the 20-foot timed walk test^10,11,41,42^, a 40-foot timed walk test^18^, a 1-min walk test^9^ or a 4-m walk test^48^. Evidence suggests that while their results are highly correlated with longer walking tests, short walking tests overestimate gait speed compared to longer walking tests such as the 6MWT^49^, and the correlation between the two types of tests is much lower in healthy middle-aged adults^50^. We tried to verify this hypothesis by comparing the results of the first minute of the 6MWT (essentially a 1-min walk test) and the average speed during the full six minutes. Our results confirm that this heterogeneity in walking tests could be a cause for conflicting results; while our final, adjusted model, showed a significant link between CRP and gait speed in females when taking the full test results, the link only bordered significance when using results from the first minute of the test. Another possible source of conflict is heterogeneity in gait measurement protocols; while some past studies asked participants to walk at their usual pace (e.g.^41,42^), others asked them to go as fast as they can (e.g.^9,18^).

Aside from allowing gait speed measurement, the 6MWT tests for physical and aerobic endurance^23,51^, and is more ecologically valid, as it resembles walking in real-life situations^23^. Age related deterioration in physical capacity in middle age is often not as accentuated as in older age, and a short walking test in usual pace might not be strenuous enough to identify a small decline in gait speed among healthy middle-aged adults. While the advantages of short walking tests in the clinical setting are clear, we recommend that to examine age-related gait speed decline in midlife in future studies, longer walking tests be used, and participants be asked to walk as fast as they can.

There are several limitations to our study. Due to the cross-sectional design, no causal relations could be inferred between inflammatory markers and gait speed decline. This is especially relevant because it is yet unclear if low-grade systemic inflammation indeed causes age related functional decline, or is simply a marker of it. Moreover, our cohort of community-residing middle-aged Muslim-Arabs was homogenous in religion and culture. While advantageous in sense of limiting covariates variability, due to similar ways of life, diet, and health behaviours, this could limit the ability to generalize this study’s findings. In future studies, a comparison between different populations could help assert the generalizability of our results. Furthermore, chronic non-communicable disease prevalence and medications were collected by self-report in our study; this bears some inaccuracy, as medications could affect several covariates included in our analyses. Finally, although we adjusted for gender, socioeconomic status and BMI, various confounders we could not account for could impact the reliability of our results.

We recommend future studies to be conducted using longitudinal settings of 5–10 years to follow-up, to help assess the directionality of the relationship between inflammation and physical capacity. We also suggest studies to measure a wider array of inflammation markers, as they could serve a variety of roles in inflammation-related physical capacity decline.

## Conclusion

In conclusion, serum CRP, but not fibrinogen levels are associated with decreased gait speed in middle-aged females, when adjusting for age, height, BMI, cardiometabolic risk factors, executive function, smoking and aerobic physical activity. Understanding the influence of inflammation on gait speed may help improve early identification, intervention, and prevention of mobility decline, and consequently promote healthy aging.

## Data Availability

The datasets used and analyzed in this study are available from the corresponding author upon reasonable request.

## References

[1] WHO (2012). Good health adds life to years. Global brief for World Health Day 2012. World Health Organ..

[2] Shumway-Cook A, Ciol MA, Yorkston KM (2005). Mobility limitations in the medicare population: Prevalence and sociodemographic and clinical correlates. J. Am. Geriatr. Soc..

[3] Ferrucci L, Cooper R, Shardell M (2016). Age-related change in mobility: Perspectives from life course epidemiology and geroscience. J. Gerontol. Ser. A.

[4] Menant JC, Schoene D, Sarofim M, Lord SR (2014). Single and dual task tests of gait speed are equivalent in the prediction of falls in older people: A systematic review and meta-analysis. Ageing Res. Rev..

[5] Dumurgier J, Artaud F, Touraine C (2017). Gait speed and decline in gait speed as predictors of incident dementia. J. Gerontol. Ser. A.

[6] Abellan Van Kan G, Rolland Y, Andrieu S (2009). Gait speed at usual pace as a predictor of adverse outcomes in community-dwelling older people an International Academy on Nutrition and Aging (IANA) task force. J. Nutr. Health Aging.

[7] Studenski S, Perera S, Patel K (2011). Gait speed and survival in older adults. JAMA.

[8] Brown PJ, Roose SP, Zhang J (2016). Inflammation, depression, and slow gait: A high mortality phenotype in later life. J. Gerontol. Ser. A.

[9] Heumann Z, Youssim I, Kizony R (2022). The relationships of fibrinogen and C-reactive protein with gait performance: A 20-year longitudinal study. Front. Aging Neurosci..

[10] Verghese J, Holtzer R, Lipton RB, Wang C (2012). High-sensitivity C-reactive protein and mobility disability in older adults. Age Ageing.

[11] Verghese J, Holtzer R, Oh-Park M (2011). Inflammatory markers and gait speed decline in older adults. J. Gerontol. Ser. A.

[12] Baptista G, Dupuy AM, Jaussent A (2012). Low-grade chronic inflammation and superoxide anion production by NADPH oxidase are the main determinants of physical frailty in older adults. Free Radic. Res..

[13] Kositsawat J, Kuo CL, Barry LC (2020). Interaction between vitamin D and interleukin 6 on slow gait speed: 6-year follow-up data of older adults from InCHIANTI. J. Gerontol. Ser. A.

[14] Ko S, Stenholm S, Metter EJ, Ferrucci L (2012). Age-associated gait patterns and the role of lower extremity strength: Results from the Baltimore Longitudinal Study of Aging. Arch. Gerontol. Geriatr..

[15] Alzaid H, Ethofer T, Kardatzki B (2022). Gait decline while dual-tasking is an early sign of white matter deterioration in middle-aged and older adults. Front. Aging Neurosci..

[16] Kuh D, Cooper R, Sattar N (2019). Systemic inflammation and cardio-renal organ damage biomarkers in middle age are associated with physical capability up to 9 years later: Findings from a british birth cohort study. Circulation.

[17] Dupont J, Antonio L, Dedeyne L (2021). Inflammatory markers are associated with quality of life, physical activity, and gait speed but not sarcopenia in aged men (40–79 years). J. Cachexia Sarcopenia Muscle.

[18] Tomey K, Sowers MF, Zheng H, Jackson EA (2009). Physical functioning related to C-reactive protein and fibrinogen levels in mid-life women. Exp. Gerontol..

[19] Stellmann JP, Neuhaus A, Götze N (2015). Ecological validity of walking capacity tests in multiple sclerosis. PLoS ONE.

[20] Tzemah-Shahar R, Hochner H, Iktilat K, Agmon M (2022). What can we learn from physical capacity about biological age? A systematic review. Ageing Res. Rev..

[21] Eden MM, Tompkins J, Verheijde JL (2017). Reliability and a correlational analysis of the 6MWT, ten-meter walk test, thirty second sit to stand, and the linear analog scale of function in patients with head and neck cancer. Physiother. Theory Pract..

[22] Du H, Wonggom P, Tongpeth J, Clark RA (2017). Six-minute walk test for assessing physical functional capacity in chronic heart failure. Curr. Heart Fail. Rep..

[23] Rikli RE, Jones CJ (1998). The reliability and validity of a 6-minute walk test as a measure of physical endurance in older adults. J. Aging Phys. Act..

[24] Harada ND, Chiu V, Stewart AL (1999). Mobility-related function in older adults: Assessment with a 6-minute walk test. Arch. Phys. Med. Rehabil..

[25] Friedlander Y, Kark JD, Sinnreich R (2006). Fibrinogen and CRP in Israeli families: Genetic and environmental sources of concentrations and longitudinal changes. Atherosclerosis.

[26] Reitan RM (1955). The relation of the Trail Making Test to organic brain damage. J Consult. Psychol..

[27] Tombaugh TN (2004). Trail Making Test A and B: Normative data stratified by age and education. Arch. Clin. Neuropsychol..

[28] Koh KK, Han SH, Quon MJ (2005). Inflammatory markers and the metabolic syndrome: Insights from therapeutic interventions. J. Am. Coll. Cardiol..

[29] Dumurgier J, Elbaz A, Dufouil C (2010). Hypertension and lower walking speed in the elderly: The Three-City study. J. Hypertens..

[30] Okoro CA, Zhong Y, Ford ES (2006). Association between the metabolic syndrome and its components and gait speed among U.S. adults aged 50 years and older: A cross-sectional analysis. BMC Public Health.

[31] Kearney FC, Harwood RH, Gladman JRF (2013). The relationship between executive function and falls and gait abnormalities in older adults: A systematic review. Dement. Geriatr. Cogn. Disord..

[32] Gordon EH, Peel NM, Samanta M (2017). Sex differences in frailty: A systematic review and meta-analysis. Exp. Gerontol..

[33] Arber S, Cooper H (1999). Gender differences in health in later life: The new paradox?. Soc. Sci. Med..

[34] Nathanson CA (1967). (1975) Illness and the feminine role: A theoretical review. Soc. Sci. Med..

[35] Santoro A, Bientinesi E, Monti D (2021). Immunosenescence and inflammaging in the aging process: age-related diseases or longevity?. Ageing Res. Rev..

[36] Yang Y, Kozloski M (2011). Sex differences in age trajectories of physiological dysregulation: Inflammation, metabolic syndrome, and allostatic load. J. Gerontol. Ser. A.

[37] Rexrode KM, Pradhan A, Manson JE (2003). Relationship of total and abdominal adiposity with CRP and IL-6 in women. Ann. Epidemiol..

[38] Kattula S, Byrnes JR, Wolberg AS (2017). Fibrinogen and fibrin in hemostasis and thrombosis. Arterioscler. Thromb. Vasc. Biol..

[39] Letcher RL, Chien S, Pickering TG (1981). Direct relationship between blood pressure and blood viscosity in normal and hypertensive subjects: Role of fibrinogen and concentration. Am. J. Med..

[40] Bohannon RW (2008). Population representative gait speed and its determinants. J. Geriatr. Phys. Ther..

[41] Kositsawat J, Barry LC, Kuchel GA (2013). C-reactive protein, vitamin D deficiency, and slow gait speed. J. Am. Geriatr. Soc..

[42] Kuo H-K, Bean JF, Yen C-J, Leveille SG (2006). Linking C-reactive protein to late-life disability in the National Health and Nutrition Examination Survey (NHANES) 1999–2002. J. Gerontol. Ser. A.

[43] Chen JL, Chen DM, Luo C (2021). Fibrinogen, fibrin degradation products and risk of sarcopenia. Clin. Nutr..

[44] Gemmati D, Varani K, Bramanti B (2019). “Bridging the Gap” everything that could have been avoided if we had applied gender medicine, pharmacogenetics and personalized medicine in the gender-omics and sex-omics era. Int. J. Mol. Sci..

[45] Black PH (2002). Stress and the inflammatory response: A review of neurogenic inflammation. Brain Behav. Immun..

[46] Graham JE, Robles TF, Kiecolt-Glaser JK (2006). Hostility and pain are related to inflammation in older adults. Brain Behav. Immun..

[47] van den Berg R, Jongbloed EM, de Schepper EIT (2018). The association between pro-inflammatory biomarkers and nonspecific low back pain: a systematic review. Spine J..

[48] Renner SW, Qiao Y, Gmelin T (2022). Association of fatigue, inflammation, and physical activity on gait speed: The Long Life Family Study. Aging Clin. Exp. Res..

[49] Dean CM, Richards CL, Malouin F (2016). Walking speed over 10 metres overestimates locomotor capacity after stroke. Clin. Rehabil..

[50] Dalgas U, Severinsen K, Overgaard K (2012). Relations between 6 minute walking distance and 10 meter walking speed in patients with multiple sclerosis and stroke. Arch. Phys. Med. Rehabil..

[51] Tuna HD, Edeer AO, Malkoc M, Aksakoglu G (2009). Effect of age and physical activity level on functional fitness in older adults. Eur. Rev. Aging Phys. Act..

